# Excerpta

**Published:** 1850-12

**Authors:** 


*Excerpta.—In certain sects of religion* there is a class known (at the south) by the soubriquet of Hard Shells—a class, whose hog-like souls no precepts of wisdom, human or divine, can lift above the mire of conceit and stupidity in which they instinctively wallow. Perhaps the practitioner of medicine has therefore no right to complain, if, *in the healing art,* a like species of hard cases are to be encountered in officious visits around the bedside of the halt, the lame and the blind—suggestions drop through their prolific brains with the facility of water through a sieve. If all such were to visit a menagerie, their asinine diathesis might be much improved.—*New Hampshire Jour.*

